# Do market shocks generate gender-differentiated impacts? Policy implications from a quasi-natural experiment in Bangladesh

**DOI:** 10.1016/j.wsif.2019.102272

**Published:** 2019

**Authors:** Khondoker Abdul Mottaleb, Dil Bahadur Rahut, Olaf Erenstein

**Affiliations:** Socioeconomics Program, International Maize and Wheat Improvement Center (CIMMYT), Carretera Mex-Veracruz, Km. 45, El Batan, Texcoco CP 56237, Mexico

**Keywords:** Expenditure, Female-headed households, Food, Impacts, Non-food, Price hikes

## Abstract

Using information collected from two rounds of household income and expenditure surveys (HIES 2005 and 2010) in Bangladesh, this study examines the gender-differentiated impacts of the commodity price hikes in 2008 on food and non-food consumption behavior based on the sex of the household head. Applying the difference-in-difference estimation method in a quasi-natural experiment setting, this study demonstrates that, in general, commodity price hikes more adversely affect female-headed households. In 2010, they reduced expenditures on food and non-food items, and particularly cereal, non-cereal, and education expenditures, more than male-headed households did. This study also shows that the impacts of commodity price hikes were lower on the female-headed households headed by educated females as well as those who owned larger pieces of land and received remittances. These subsets were not affected by the commodity price shocks as examined in 2010. The findings strongly suggest that the provision of both human and physical capital is instrumental in developing countries to empower female-headed households to enhance their buffering capacity to withstand economic shocks.

## Highlights

• This study demonstrates gender differentiated impacts of commodity price hikes.
• Market shock generated gender differentiated impacts on female-headed households.
• Female heads human and physical capital were not severely adversely affected.
• Access to remittance income also reduced the adverse impacts of market shocks.
• Female heads should be equipped with more human and physical capital to buffer against market shocks.

## 1 Introduction

It is increasingly recognized that the global economic downturn and the unprecedented commodity price hikes in 2008 led to an increase in the absolute numbers of impoverished people globally (FAO, 2008, FAO, 2011; Harttgen & Klasen, 2012). For example, due to the increase in the price of food baskets in Bangladesh and Ethiopia, the incidence of rural poverty has increased in both countries (Balagtas, Bhandari, Cabrera, Mohanty, & Hossain, 2014; Gelaw & Sileshi, 2013). Between 2006 and 2008, due to the hikes in commodity prices, the producers' prices of maize increased by 51%, from USD 250/ton to USD 376/ton; the rice price increased by 35%, from USD 394/ton to USD 531/ton, and the wheat price increased by 65%, from USD 206/ton to USD 340/ton (FAO, 2019). At present, global commodity prices have remained relatively high compared to pre-2008 levels (FAO, 2019).

The extent of the fall-out of the commodity price hike remains to be fully understood. For instance, did the commodity price hike generate gender-differentiated impacts? Did female-headed households respond differently than male-headed households in relation to food and non-food expenditures in the face of these price hikes? A clearer understanding of these issues is important to ensure the welfare of female-headed households in developing countries and to work towards achieving gender parity as per the Sustainable Development Goals (SDGs) of the United Nations (UN) by 2030. Unfortunately, only a few empirical studies address this issue (e.g., Baker, 2008; Compton, Wiggins, & Keats, 2010; FAO, 2008; FAO and WFP, 2008; Kumar & Quisumbing, 2013; Quisumbing et al., 2008; Quisumbing, Kumar, & Behrman, 2018).

There is a consensus in the literature that in general, female-headed households are in the poorest-of-the-poor group in developing countries (Bastos, Casaca, Nunes, & Pereirinha, 2009; Elmelech & Lu, 2004; Fuwa, 2000; Mallick & Rafi, 2010; World Bank, 2012). Women in developing countries are mostly excluded from inheriting the land and land ownership, despite existing national laws on women's rights to land (Kieran, Sproule, Doss, Quisumbing, & Kim, 2015; Quisumbing, 1996; World Bank, 2012; World Bank, FAO and IFAD, 2009). For example, women in Bangladesh own significantly fewer agricultural production assets than men (Quisumbing, Haddad, & Peña, 2001; Quisumbing, Roy, Njuki, Tanvin, & Waithanji, 2013; Quisumbing & Maluccio, 2003), and have less access to information and agricultural extension services (Quisumbing & Pandolfelli, 2010), and are more socially constrained (Seymour, 2017).

Poor households, in general, spend proportionally more on food items. Consequently, one may expect the welfare loss of the female-headed poor households due to the commodity price hikes to be higher than the loss of the male-headed households (Campos & Garner, 2014; FAO, 2008; Kumar & Quisumbing, 2013; Quisumbing et al., 2018). Kumar & Quisumbing et al. (2013) use Ethiopia as a case to demonstrate the effects of the commodity price hikes in 2007–08. They show that female-headed households in Ethiopia experienced price shocks more frequently; were more vulnerable to the shocks, and were less able to recover their welfare losses than male-headed households were. This is mainly because of the pre-crisis inequality in access to land and other resources. Compton et al. (2010) demonstrate that among the poor and marginal food consumption groups, female-headed households were 1.6 times more food insecure than male-headed households due to the commodity price hikes in 2007–08. Unequal access to land and other resources including agricultural extension services are the major causes behind the gender-differentiated impacts of the commodity price hikes on a household's welfare (Quisumbing et al., 2018; Quisumbing & Maluccio, 2003).

**Fig. 1:**
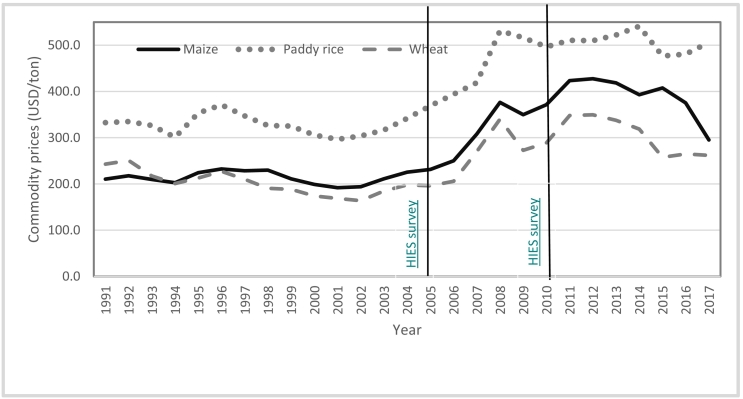
International prices (USD/ton) of wheat, maize and paddy rice, 1991–2017. Source: FAO (2019).

To date few studies have assessed the gender-differentiated fall-out of the commodity price hikes in 2008 on food and non-food expenditures and mitigating factors: e.g., the influence of physical capital, such as land and agricultural equipment ownership; and the influence of remittance income and labor allocation to the non-farm sector. Note that such welfare impacts of price hikes on female-headed households are likely to be heterogeneous across countries given the heterogeneous social status of women and associated access to both physical and human capitals. It necessitates conducting country-specific case studies on the gender-differentiated impacts of the commodity price hikes. Using information collected from more than 22,000 households by the Bangladesh Bureau of Statistics (BBS) in 2005 and 2010 (BBS, 2012a, BBS, 2012b) and applying the difference-in-difference estimation procedure, this study examines the impacts of commodity price hikes on the expenditure on food and non-food items by the households based on the sex of the household head in Bangladesh. As the commodity prices spiked in 2008,^1^ the present study treats the 2005 data sets as ‘before the shock’ and the 2010 data sets as ‘after the shock’ (Fig. 1).

The case of Bangladesh is worth investigating for several reasons. First, it is widely documented that, women in Bangladesh own significantly fewer agricultural production assets than men (Quisumbing et al., 2001; Quisumbing & Maluccio, 2003; Sraboni, Malapit, Quisumbing, & Ahmed, 2014; The Economist, 2013) and in general, have less access to information and agricultural extension services than men (Quisumbing & Pandolfelli, 2010); and are more socially constrained (Seymour, 2017). Despite the recent changes in the participation of women in the formal labor force in Bangladesh (Mottaleb & Sonobe, 2011), in many cases, the religious and social norms restrict women's participation. Particularly, rural women in Bangladesh mostly contribute unpaid family labor and engage in jobs within the house or adjacent areas to their homestead (Kabeer, Mahmud, & Tasneem, 2011), such as post-harvest activities (Hossain, Bose, & Ahmad, 2004; Zaman, 1995). Some recent changes, however, can be observed in women's participation in the formal labor market, as young women from poor families are increasingly migrating to (semi-)urban areas mainly to work inter alia as garment industry workers (Mottaleb & Sonobe, 2011). Also, the participation of women from poor families in agricultural labor has increased significantly (Jaim & Hossain, 2011; Rahman, 2010; World Bank, 2012).

Second, within South Asia, Bangladesh's recent achievement in reducing the gender gap in social, political, cultural, and educational attainments is remarkable. Currently, Bangladesh is ranked 48th among 149 countries in terms of gender parity based on economic participation and opportunity; educational attainment; health and survival, and political empowerment (World Economic Forum, 2018). Compared to Bangladesh, Sri Lanka ranked 100, Nepal 105, India 108, and Pakistan 148 (World Economic Forum, 2018). Bangladesh ranks best in terms of the gender gap index among Islamic as well as South Asian countries (World Bank, 2012). The average number of children per woman has decreased to two from seven in Bangladesh; girls' school enrollment has increased dramatically; and, since 1990, female labor force participation has doubled (World Bank, 2012). Importantly, mainly to delay motherhood and adolescent girls' marriage and to increase the female students' school attainment, the government of Bangladesh, with the World Bank and Asian Development Bank, initiated the Female Secondary School Assistance Project (FSSAP) in 1994. Under this program, tuition-free education, book allowances, a monthly stipend, and free secondary school examination are provided to every female student in grades 6–10 (Mottaleb, Mohanty, & Mishra, 2015). It is against this backdrop that we examine the impacts of education and socio-economic status of females on their food and non-food expenditure behavior under the commodity price hikes regime.

One of the novelties of the present study is that this is the first attempt to quantitatively examine the impacts of market shocks on the food and non-food expenditure behavior of female-headed households, controlling for the pre-existing socio-economic conditions, such as land ownership. To our knowledge, only two studies of Balagtas et al. (2014) and Mallick and Rafi (2010) are distantly related to our study. Balagtas et al. (2014) use Bangladesh as a case to show that the commodity price hikes in 2007/08 pushed back an additional 13 million people below the poverty line, but did not consider the gendered differentiated impacts of the commodity price hikes. Mallick and Rafi (2010), on the other hand, applying the Ordered Probit estimation procedure, demonstrate that in general male-headed households are 37% more food secure than female-headed households; but not address market shocks in Bangladesh. Considering these facts, our study fills a knowledge gap in gender studies literature.

Importantly, although the present study focuses on Bangladesh, the issues that are examined in this study are closely replicated in millions of households across South Asia; where similar to Bangladesh, the socio-economic and the educational status of women, in general, is lower than men. This generalizability of the status of the female in South Asian society highlights the potential policy relevance of the present study. The study is organized as follows: Section 2 presents data sources and descriptive findings; Section 3 specifies the difference-in-difference estimation procedure applied in this study; Section 4 presents the econometric findings and Section 5 presents the conclusions and policy implications.

## 2 Data and setting

### 2.1 Data

This study uses data from two successive Household Income and Expenditure Surveys (HIES) conducted in 2005 and 2010 by the Bangladesh Bureau of Statistics (BBS, 2012b, BBS, 2012a). After independence in 1971, the BBS conducted the first round of HIES in 1973–74. Between then and 2010, the BBS successfully conducted 15 rounds of HIES, but information on consumption was not included until the 2000 round. In HIES 2000, BBS started including detailed information on household incomes, and particularly, expenditures on food and non-food items. The expenditure on food and non-food items includes both in-kind expenditures in the form of the monetary value of the self-produced item and non-farm food bought from the market. With the financial and technical support of the World Bank, the BBS uses a two-stage stratified random sampling process in which, in the first stage, the BBS selects Primary Sampling Units (PSUs) consisting of specific geographical areas in both rural and urban areas, and in the second stage, randomly selects 20 households from each PSU.

The HIES 2005 survey included 504 PSUs and 10,080 households randomly selected from 8 divisions, 64 districts and 364 sub-districts. The HIES 2010 survey randomly selected 1000 PSUs and 12,240 households from all nine administrative divisions, 64 districts and 372 sub-districts. Out of the aggregate 22,320 sampled households across two waves, nine households were dropped for lack of information on food and non-food expenditures. The present study is thus based on information collected from 22,311 households in 2005 and 2010, of which 9036 were from urban areas, and 13,275 were from rural areas; females headed 7789 households and males headed 19,522.

### 2.2 Descriptive findings

**Fig. 2:**
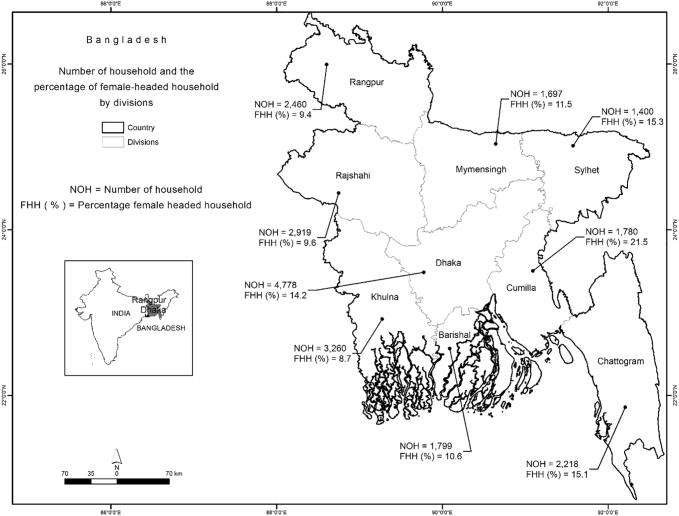
Sample size (# of households, NOH) and share of female-headed households (FHH, %) by division, Bangladesh. Sources: Bangladesh Bureau of Statistics (BBS): HIES 2005 and HIES 2010.

In Bangladesh, the eldest male or female earner is considered the head of the household (BBS, 2007). However, in the case where the main earner works as a migrant worker, the spouse can be the household head even though he or she may not be directly involved in income generating activities. The distribution of the 22,311 sampled households and the share of female-headed households in the seven administrative divisions of Bangladesh is presented in Fig. 2. Dhaka (13.5%), Chittagong (17.9%) and Sylhet (15.3%) divisions stand out for having relatively more female-headed households. This is linked to the pervasiveness of overseas male migrant workers from these divisions, as mainly male family members go abroad, leaving the other family members in the country. In 2013, there were 1.53 million foreign remittance recipient households in Bangladesh, of which nearly 33% were from Dhaka division, and 30% were from Chittagong division (BBS, 2014). Sylhet is well known in Bangladesh for sending migrant workers particularly to the United Kingdom (UK).

Descriptive statistics of the sample households are presented in Table 1. On average, more than 25% of the total sampled households received remittances amounting to Bangladesh Taka (BDT) 11,540 annually.^2^ On average, a sample household had 0.69 acres of land,^3^ and the value of the agricultural equipment and assets owned by each household was worth BDT 2320. In HIES 2005 and 2010, the monetary value of 19 different agricultural machinery and assets were aggregated into the value of agricultural asset ownership. The average household head was 45 years old with 3.73 years of formal schooling and the average years of schooling of their spouse was nearly three years. The average household consisted of 4.81 family members. Table 1 shows that 63% of the sampled households allocated a portion of their labor to the non-farm sector, and nearly 62% of the sampled households were from rural areas.

**Table 1:** Characteristics of the sampled household over the years by the sex of the household head.

Household headed by	All	Female		Male		Mean difference (male – female)^a^	
Sample year		2005	2010	2005	2010	2005	2010
No. of households	22,311	1040	1749	9032	10,490		
% Received remittances	25.12 (43.37)	53.37 (49.91)	55.46 (49.72)	25.83 (43.77)	16.64 (37.25)	−27.53^⁎⁎⁎^ (−18.91)	−38.82^⁎⁎⁎^ (−38.26)
Remittance received (‘000, BDT)	13.39 (69.94)	30.18 (64.48)	59.26 (173.63)	5.94 (25.93)	10.48 (62.87)	−24.24^⁎⁎⁎^ (−23.03)	−48.77^⁎⁎⁎^ (−21.52)
Land operated (acres)	0.66 (1.41)	0.30 (1.07)	0.28 (0.80)	0.76 (1.58)	0.69 (1.34)	0.46^⁎⁎⁎^ (9.15)	0.41 (12.39)
Value of agricultural assets owned (‘000, BDT)	2.78 (21.18)	0.21 (1.94)	0.91 (6.70)	1.64 (14.59)	4.32 (27.53)	1.43^⁎⁎⁎^ (3.15)	3.41^⁎⁎⁎^ (5.16)
Age of the household head	45.74 (13.72)	46.61 (14.03)	46.37 (14.70)	45.27 (13.44)	45.95 (13.74)		
Years of schooling of the household head	3.83 (4.48)	2.15 (3.50)	2.68 (3.84)	3.99 (4.51)	4.05 (4.56)	1.84^⁎⁎⁎^ (12.72)	1.38^⁎⁎⁎^ (11.93)
No. of family members	4.68 (1.98)	3.53 (2.02)	3.44 (1.80)	5.01 (2.03)	4.73 (1.84)	1.48^⁎⁎⁎^ (22.31)	1.29^⁎⁎⁎^ (27.22)
Years of schooling of the spouse	3.16 (3.98)	2.55 (3.80)	2.85 (3.98)	3.03 (3.92)	3.38 (4.03)		
% Households allocated labor for non-farm economic activities	63.36 (48.18)	45.48 (49.82)	38.54 (48.68)	66.31 (47.27)	66.73 (47.12)	20.8^⁎⁎⁎^ (13.38)	28.2^⁎⁎⁎^ (23.06)
% Of rural households	59.50 (49.09)	62.88 (48.33)	63.46 (48.17)	59.59 (49.07)	58.43 (49.29)		

Sources: Bangladesh Bureau of Statistics (BBS). HIES 2005 and HIES 2010. Numbers in parentheses are standard errors.

^*^Significant at the 10% level; ^**^Significant at the 5% level; ^***^Significant at the 1% level.

a Numbers in parentheses are t-statistics.

Compared to male-headed households, the percentage of remittance-receiving households was higher among the female-headed households, and the female-headed households received higher remittances in both the sample years (Table 1). Across the sample years, female-headed households had fewer years of schooling, fewer family members and less agricultural equipment compared to male-headed households (Table 1). Female-headed households were less likely to allocate labor to the non-farm sector than male-headed households were, and were more likely to reside in rural areas. These findings generally support previous research showing that women in Bangladesh owned significantly fewer productive agricultural assets and less land than men (Quisumbing et al., 2013; Quisumbing & Maluccio, 2003). In the econometric estimation procedure, this study explicitly includes pre-existing situations to examine their influence on the expenditure behavior of female-headed households under market price shocks compared to male-headed households.

The expenditure pattern of the sample households over the years are presented in Table 2 is in per capita yearly basis. On average across 2005 and 2010, a sampled household spent BDT 19,529 on food items per capita annually, split into BDT 11,768 for non-cereal food items (such as fish, meat, vegetables, oil and so on), and BDT 7760 on cereals (such as rice, wheat, puffed rice, and flour). On average, a member of a sampled household consumed 266 kg of cereals annually in which rice is the principal cereal item. On average, a sampled household spent per capita annually BDT 11,332 on non-food items, including BDT 884.5 on health-related expenditures and BDT 1116.3 on education.

**Table 2:** Yearly consumption expenditure of food and non-food items per capita/year by the sampled household over the years and by the sex of the household head.

Household headed by	All	Female		Male		Mean difference (male – female)^a^	
Sampled year		2005	2010	2005	2010	2005	2010
Expenditure on all food items (‘000, BDT)	19.53 (16.51)	7.84 (5.14)	30.64 (19.21)	7.33 (3.69)	29.34 (15.67)	−0.50^⁎⁎⁎^ (−3.98)	−1.30^⁎⁎⁎^ (−3.11)
Expenditure on non-cereal food items (‘000, BDT)	11.77 (12.63)	4.72 (4.29)	19.14 (15.90)	4.23 (3.19)	17.73 (13.72)	−0.48^⁎⁎⁎^ (−4.45)	−1.41^⁎⁎⁎^ (−3.89)
Expenditure on cereal food items (‘000, BDT)	7.76 (5.25)	3.12 (1.27)	11.50 (5.19)	3.10 (0.99)	11.61 (3.89)	−0.02 (−0.57)	0.11 (1.00)
Cereal consumption (kg)	266.18 (127.08)	173.03 (68.94)	337.21 (142.57)	173.94 (55.34)	342.98 (113.71)	0.91 (0.49)	5.77^⁎^ (1.89)
Expenditure on non-food items (‘000, BDT)	11.33 (17.51)	9.30 (16.76)	16.52 (22.76)	7.21 (10.84)	14.22 (20.22)	−2.09^⁎⁎⁎^ (−5.49)	−2.30^⁎⁎⁎^ (−4.34)
Expenditure on health (BDT)	1116.34 (3957.30)	617.12 (2104.59)	1673.98 (3805.05)	567.18 (1574.33)	1545.69 (5272.85)	−4.46 (−0.08)	−177.31^⁎^ (−1.56)
Expenditure on education (BDT)	884.55 (3466.33)	511.34 (1053.31)	1346.94 (4087.89)	506.88 (1691.28)	1169.63 (4466.04)	−49.94 (−0.93)	−128.30 (−0.98)

Sources: Bangladesh Bureau of Statistics (BBS). HIES 2005 and HIES 2010.

^*^Significant at the 10% level; ^**^Significant at the 5% level; ^***^Significant at the 1% level.

a Numbers in parentheses are t-statistics.

Strikingly, female-headed households had higher expenditures per capita on food and non-food items in 2005; and the differences widened in 2010 (Table 2). In 2005, on average, a female-headed household annually spent BDT 500 more on food items and BDT 2090 more on non-food items per capita. In 2010, these differences further widened to BDT 1300 and BDT 2300 respectively. Expenditure on non-cereal food items was also higher in the case of a female-headed household in both 2005 and 2010. Cereal consumption, as well as yearly per capita expenditure on cereal food, health and education were similar for female- and male-headed households in the base year 2005. However, in 2010, female-headed households consumed on average, 6 kg less cereal yearly per capita, but spent BDT 177 more on health issues than male-headed households.

One might be wondering why female-headed households had higher food and non-food per capita expenditure compared to male-headed households. This is probably because of the economies of scale available to larger household sizes, given that the average number of family members in female-headed households was significantly lower than male-headed households (Table 1). Three independent observations (Deaton & Paxson, 1998; Koohi-Kamali, 2008; Vernon, 2005) confirmed the influence of family size on poverty due to the availability of economies of scale in consumption. Particularly, Vernon, (2005) confirmed that doubling the size of a household reduces per capita food expenditure by 30%. In addition, with more family members, male-headed households may pay lower prices for similar foods, because they can exploit the benefits of bulk purchasing, compared to smaller female-headed households.

## 3 Model specification

The discussion in the previous section indicates that, in addition to pre-existing conditions, such as heterogeneous access to land and other productive assets by sex, commodity prices and household sizes can generate gender-differentiated impacts. In addition, female-headed households, on average receive more remittances than male-headed households (Table 1). The poverty reducing impacts of remittance and the impacts of remittances on the households' ability to absorb price shock is widely recognized in the literature (Adams Jr & Page, 2005; Taylor, Mora, Adams, & Lopez-feldman, 2005).

We applied the difference-in-difference (DID) estimation approach to examine the impacts of food price hikes in 2008 on food and non-food expenditures of female and male-headed households. Econometrically, to disaggregate the effects of the commodity price hikes from the other effects on expenditure behavior of female-headed households compared to the male-headed households, the equation is specified as:

(1) $$Y_{\mathrm{DiT}}=\beta_{0}+\beta_{1}\left(\mathrm{DY}2010\right)+\beta_{2}{\left(\mathrm{DFHH}\right)}_{i}+\beta_{3}\left({\mathrm{DFHH}}_{i}*\mathrm{DY}2010\right)+\left(Z_{i}\right)\theta_{i}+\xi_{\mathrm{it}}$$

where *Y*_*DiT*_ is a vector of dependent variables that includes yearly total expenditure on non-food items and, separately, expenditure on health and education; expenditure on total food items; and separately on cereal and non-cereal food items. *DFHH*_*i*_ is the dummy for a treatment household that assumes a value of 1 if a household is headed by a female or 0 otherwise. *DY2010* is the dummy variable for the year 2010 that assume a value of 1 if the year is 2010, and 0 for the year 2005, which is the base. The *Z*_*i*_ is a vector of variables that include: the average cereal price (BDT/kg) calculated at the household level; age of the household head, the number of family members, a rural household dummy that assumes a value of 1, if a household is located in a rural area, and 0 otherwise; a non-farm labor allocation dummy that assumes a value of 1, if a household allocates a portion of its labor to the non-farm sector, or 0 otherwise; the size of land operated in acres; a dummy for a remittance-recipient household that assumes a value of 1 if a household received remittances, and 0 otherwise; and the monetary value of the agricultural equipment and assets owned by the household. The vector *Z*_*i*_ includes a series of multiplicative dummies, which were generated by multiplying the female-headed household dummy with year dummies and the variables of interests, such as years of schooling, the monetary value of the agricultural equipment and assets, and remittance recipient household dummy, and the size of the land holdings. These multiplicative dummies are supposed to capture the heterogeneous impacts of commodity price shocks on the female-headed households in year 2010. *β*_0_ is a scalar parameter; *β*_*i*_ and *θ*_*i*_ are the parameters to be estimated; *i* stands for individual i; t stands for year (*t* = 2005, 2010); and *ξ* is the random error term. From Eq. (1):

$$\hat{\beta_{0}}=\bar{E}\left(y\right|\mathrm{Year}=2005,\;\mathrm{DFHH}=0)$$

$$\hat{\beta_{1}}=\bar{E}\left(y\right|\mathrm{Year}=2010,\;\mathrm{DFHH}=0)-\left(y|\mathrm{Year}=2005,\mathrm{DFHH}=0\right)$$

$$\hat{\beta_{2}}=\bar{E}\left(y\right|\mathrm{Year}=2010,\;\mathrm{DFHH}=1)-\left(y|\mathrm{Year}=2005,\mathrm{DFHH}=0\right)$$

$$\hat{\beta_{3}}=\bar{E}\left[\left(y\right|\mathrm{Year}=2010,\;\mathrm{DFHH}=1\right)-\left(y|\mathrm{Year}=2005,\mathrm{DFHH}=1\right)]-\bar{E}\;[\left(y|\;\text{year}=2010,\mathrm{DFHH}=0\right)-(\left(y|\;\text{year}=2005,\mathrm{DFHH}=0\right)]$$

Essentially, $\hat{\beta_{3}}$ is equal to (DFHH_(10)_ -DFHH _(05)_) - (MHD_(10)_ - MHH _(05)_), which is the DID estimator, where MHD is the dummy for a male-headed household.

Note that as the HIES datasets are cross-sectional, the application of the conventional panel data estimation procedure by applying a Fixed-effect or Random-effect estimation procedure is not possible. However, taking the opportunity of the repeated presence of at least 461 sub-districts in two surveys, this study formed a pseudo-panel at the sub-district level and applied the sub-district level Fixed-effect estimation procedure in estimation Eq. (1).

## 4 Econometric findings

Table 3 presents the estimated functions applying a sub-district level Fixed-effect estimation procedure explaining food and non-food expenditures, with separate functions for cereal and non-cereal food expenditures. Compared to the base in 2005, households in 2010, in general, spent more on food and cereal food in particular, and less on non-food items. Probably with the decrease in real income of the sampled households in the face of the increase in commodity prices, households first reduced the consumption of non-food items, to ensure the minimum food requirements per capita. In addition, in 2010, the sampled households probably spent more on cereal food items, because cereals are relatively more affordable than costly non-cereal food items, and by switching to cereals, households could ensure the required calorie intake.

**Table 3:** Estimated functions applying the Fixed-Effect estimation procedure explaining per capita yearly expenditure on different food and non-food items at the household level in Bangladesh.

Dependent variables	All food expenditure (‘000 BDT)	Expenditure on non-cereal food items (‘000, BDT)	Expenditure on cereal food items (‘000 BDT)	All non-food expenditure (‘000 BDT)	Education expenditure (‘000, BDT)	Medical expenditure (‘000, BDT)
Year 2010 (DY2010, dummy, yes = 1)	3.67⁎⁎ (1.78)	−1.34 (1.52)	5.00⁎⁎⁎ (0.35)	−6.86⁎⁎⁎ (1.98)	−0.97 (0.60)	0.027 (0.16)
Female-headed household (DFHH, dummy, yes = 1)	1.82⁎⁎⁎ (0.39)	2.05⁎⁎⁎ (0.34)	−0.23⁎⁎ (0.10)	1.26 (1.49)	0.38⁎⁎⁎ (0.11)	0.14⁎ (0.08)
DFHH x DY2010	−2.65⁎⁎⁎ (0.80)	−2.92⁎⁎⁎ (0.65)	0.26 (0.27)	−0.093 (1.78)	−0.62⁎⁎⁎ (0.17)	−0.042 (0.14)
Age of the household head	0.077⁎⁎⁎ (0.01)	0.052⁎⁎⁎ (0.01)	0.026⁎⁎⁎ (0.00)	0.099⁎⁎⁎ (0.01)	0.017⁎⁎⁎ (0.00)	0.012⁎⁎⁎ (0.00)
Rural household (dummy, yes = 1)	−0.81⁎⁎ (0.40)	−1.18⁎⁎⁎ (0.37)	0.37⁎⁎⁎ (0.10)	−2.78⁎⁎⁎ (0.50)	−0.32⁎⁎⁎ (0.10)	−0.11⁎ (0.07)
Household allocates labor to non-farm works (DNFH, dummy, yes = 1)	−0.64⁎⁎⁎ (0.21)	−0.26 (0.18)	−0.38⁎⁎⁎ (0.05)	−0.18 (0.27)	−0.032 (0.04)	−0.047 (0.04)
DNFH x DFHH	−0.16 (0.43)	−0.57 (0.37)	0.40⁎⁎⁎ (0.10)	−0.23 (1.07)	−0.25⁎ (0.13)	−0.072 (0.09)
DNFH x DFHH x DY2010	−1.60⁎ (0.87)	−0.75 (0.72)	−0.85⁎⁎⁎ (0.27)	−1.05 (1.52)	0.16 (0.20)	−0.076 (0.17)
Years of schooling of the household head (YSH)	0.34⁎⁎⁎ (0.03)	0.36⁎⁎⁎ (0.02)	−0.023⁎⁎⁎ (0.01)	0.86⁎⁎⁎ (0.04)	0.14⁎⁎⁎ (0.01)	0.045⁎⁎⁎ (0.00)
YSH x DFHH	−0.18⁎⁎⁎ (0.06)	−0.23⁎⁎⁎ (0.05)	0.046⁎⁎⁎ (0.01)	−0.13 (0.17)	−0.00067 (0.04)	−0.025⁎ (0.01)
YSH x DFHH x DY2010	0.52⁎⁎⁎ (0.14)	0.59⁎⁎⁎ (0.11)	−0.068⁎ (0.04)	0.54⁎⁎ (0.25)	0.18⁎⁎⁎ (0.07)	0.020 (0.04)
Value of agricultural assets owned (AGAS, ‘000, BDT)	0.029⁎⁎⁎ (0.01)	0.023⁎⁎⁎ (0.01)	0.0060⁎⁎⁎ (0.00)	0.032⁎⁎⁎ (0.01)	−0.00015 (0.00)	0.0026⁎⁎ (0.00)
AGAS x DFHH	−0.33⁎⁎⁎ (0.06)	−0.23⁎⁎⁎ (0.07)	−0.10⁎⁎⁎ (0.02)	−1.52 (1.06)	0.0016 (0.02)	−0.020 (0.02)
AGAS x DFHH x DY2010	0.32⁎⁎⁎ (0.09)	0.21⁎⁎ (0.09)	0.11⁎⁎⁎ (0.02)	1.48 (1.06)	0.00029 (0.02)	0.0065 (0.02)
Land operated (LAND, acres)	0.65⁎⁎⁎ (0.12)	0.46⁎⁎⁎ (0.10)	0.20⁎⁎⁎ (0.03)	0.73⁎⁎⁎ (0.13)	0.083⁎⁎⁎ (0.02)	0.043⁎⁎ (0.02)
LAND x DFHH	0.089 (0.19)	0.16 (0.17)	−0.068 (0.04)	5.51 (4.09)	−0.065 (0.04)	0.037 (0.06)
LAND x DFHH x DY2010	1.20⁎⁎ (0.51)	0.88⁎⁎ (0.40)	0.32⁎⁎ (0.15)	−4.41 (4.14)	0.11 (0.11)	0.051 (0.10)
Remittance-receiving household (DRRH, dummy, yes = 1)	1.24⁎⁎⁎ (0.31)	1.03⁎⁎⁎ (0.28)	0.21⁎⁎⁎ (0.06)	2.15⁎⁎⁎ (0.36)	0.016 (0.06)	0.34⁎⁎⁎ (0.06)
DRRH x DFHH	−1.20⁎⁎ (0.47)	−1.17⁎⁎⁎ (0.43)	−0.027 (0.10)	1.82⁎ (1.01)	0.19 (0.13)	−0.17 (0.11)
DRRH x DFHH x DY2010	3.60⁎⁎⁎ (1.02)	3.55⁎⁎⁎ (0.85)	0.045 (0.30)	−0.030 (1.61)	0.097 (0.25)	0.35 (0.23)
Average cereal price BDT/kg	1.12⁎⁎⁎ (0.11)	0.91⁎⁎⁎ (0.10)	0.21⁎⁎⁎ (0.02)	0.86⁎⁎⁎ (0.13)	0.12⁎⁎⁎ (0.04)	0.041⁎⁎⁎ (0.01)
Constant	−17.5⁎⁎⁎ (2.24)	−15.5⁎⁎⁎ (1.94)	−1.98⁎⁎⁎ (0.44)	−15.6⁎⁎⁎ (2.64)	−2.78⁎⁎⁎ (0.82)	−1.00⁎⁎⁎ (0.28)
No. of observations	22,311	22,311	22,311	22,311	22,311	22,311
F(21, 435)	210.64	102.08	595.54	52.33	31.24	13.49
Prob > F	0.00	0.00	0.00	0.00	0.00	0.00
sigma_u	3.92	3.77	0.97	4.56	0.91	0.55
sigma_e	10.15	8.59	2.85	14.96	3.70	3.41
Rho	0.13	0.16	0.10	0.08	0.06	0.03

Source: Authors' estimation.

Note: Numbers in parentheses are robust standard errors calculated clustering observations at the sub-district level.

⁎ Significant at the 10% level.

⁎⁎ Significant at the 5% level.

⁎⁎⁎ Significant at the 1% level.

It shows that while in general, per capita yearly food and non-food expenditure and expenditure on non-cereal food, education, and health were higher, expenditure on both foods, and non-cereal food items, and education expenditure by the female-headed households in 2010 were significantly lower than the male-headed households were. *β*_3_ is the DID estimator, in 2010, a female-headed household compared to a male-headed household, on average, had:

• Lower total food expenditure by BDT 2650 per capita annually, including a decrease of BDT 2920 for non-cereals;
• Lower education expenditure by BDT 620 per capita.

The findings indicate that, in general, female-headed households were more negatively affected by the commodity price shocks than their male counterparts. Due to the price shock, female-headed households reduced non-cereal food and education expenditures in 2010.

Table 3 presents the heterogeneous impacts of commodity price shocks based on the pre-existing socio-economic conditions of female-headed households. A household head with more years of schooling spent more than others on all food and non-food items and particularly on education and health. A relatively more educated household head spent more on non-cereal food items, such as meat fish, eggs and vegetables, and importantly spent less on cereal food items. In general, higher education of the household head translates into a higher and more lucrative income, mostly out of the farm sector as higher-income households are in general spending less on cereals relative to non-cereal food expenditure yearly per capita (Table 3). Importantly, Table 3 demonstrates that in 2010, relatively highly educated female-headed households spent significantly more on all non-food items, particularly on education, and on food items, especially non-cereal food items. These findings demonstrate the importance of education in general and particularly for females to enhance the market-related shock absorption capacity of the female-headed households.

Findings show that the value of the agricultural assets owned by the sampled households had positive impacts on both food and non-food expenditures (Table 3); and even more so in the case of female-headed households, similar to the findings of Doss (2006). Although in Bangladesh the ownership of agricultural assets and machinery by female-headed households is low, the agricultural asset ownership by female-headed households still enhances their well-being. The agricultural asset ownership did generate significant positive impacts in 2010 and allowed spending more on food items, including both cereal and non-cereals. The finding indicates that the shock absorption capacity of the female-headed households can be enhanced through the provision of the ownership of productive agricultural assets.

In general, the households that are associated with the non-farm sector by allocating a portion of their labor were not better off than other households, as is reflected by the significantly lower level of per capita yearly food expenditure (Table 3). Although in general, the non-farm labor allocation is positively associated with cereal food expenditure, it did not pay off for the female-headed households in 2010. This is probably because, for female-headed households, most of the non-farm work available to them was in the form of being a day laborer in the non-farm sector. Affiliation to the non-farm sector may thus have the limited shock-absorbing capacity, particularly when female-headed households in Bangladesh own the non-farm businesses. However, the status of the non-farm sector must be considered before drawing any strong conclusions.

In general, the households that cultivated more land, spent more on both food and non-food items, and in each of the differentiated expenditure categories. Interestingly, although the size of the land utilized by female-headed households did not influence food and non-food expenditures in general, the influence was markedly positive in 2010, particularly for food, and for both cereal and non-cereal expenditures. Ensuring female land ownership can thus reduce the economic vulnerability of female-headed households due to market volatility in developing countries.

Finally, Table 3 shows that in general the remittance-recipient households spent more on food and non-food items, and in each of the differentiated food expenditure categories. Whereas in general, female-headed households receiving remittances had lower food expenditures (and lower non-cereal expenditures), they spent markedly more on food items in 2010 (including on non-cereals). This finding indicates that access to remittance income can enhance the shock-absorbing capacity of female-headed households. Overall, in line with other findings (Doss, 2006; Hoddinott & Haddad, 1995; Quisumbing & Maluccio, 2003; Sraboni et al., 2014), this study demonstrates that when female-headed households have more control over resources, the households achieve higher levels of well-being and a higher level of market shock absorbing capacity.

It is important to mention here is that HIES datasets are cross-sectional in nature. To examine the issue further, future research should use a nationally representative longitudinal dataset in which it is possible to control for the household level fixed effect. Furthermore, to fully comprehend the gender-differentiated impacts of price shocks, future research should apply the Exogenous Switching Treatment Regression estimation procedure following Aryal, Mottaleb, and Rahut (2018).

## 5 Conclusion and policy implications

Female-headed households are the poorest-of-the-poor in developing countries and, in general, poor households spend a higher percentage of their income on food items than groups that are better off. Consequently, female-headed households are likely to have higher welfare losses due to commodity price hikes than male-headed households. Using information collected from more than 22,000 households in Bangladesh and applying the difference-in-difference estimation procedure, this study presented the gender-differentiated impacts of commodity price hikes on the food and non-food expenditures by households in Bangladesh. In the face of higher commodity prices, the female-headed households, in general, reduced their food and non-food expenditure more compared to the male-headed households in 2010. The findings of this study thus show that, in general, the impacts of the 2008 commodity price hikes generated more negative impacts on the female-headed households than the male-headed households.

This study, however, also demonstrates that the conditional impacts of commodity price hikes on the welfare of the female-headed households are highly heterogeneous based on the socio-economic conditions of the female, such as education of the female-household head, the size of the land operated, agricultural asset ownership and remittance income. This study shows that the female-headed households that were headed by relatively better-educated heads, owned more agricultural assets, operating more land and receiving more remittance income, were to a large extent buffered and their expenditure on food and non-food items was less affected at the time of the commodity price hikes.

Knowing which factors enhance the buffering capacity to market shocks of the female-headed households in developing countries, can help in designing appropriate policy interventions to promote gender parity as per the SDGs of the UN. Based on the findings, this study strongly suggests the provision of general education for all in developing countries. As education enhances the capability of a person to deal with disequilibria (Schultz, 1975), the provision of general education can enhance the capacity of resource-poor households in developing countries to perform better even in unfavorable conditions brought about by market volatility. In the case of older generations, this may call for the provision of practical need-based training. Based on the findings, this study also calls for ensuring access to land and agricultural assets by females in developing countries, to enhance the shock-absorbing capacity of the female-headed households caused by market volatility. This study also suggests removing barriers to the inflow of remittance income for female-headed households in developing countries. Furthermore, other studies show that social protection (Campos, 2015) and microcredit (Hashemi, Schuler, & Riley, 1996; Islam & Maitra, 2012) can also play important roles in empowering women. Extending the reach and strengthening the social inclusiveness of such programs may thereby further complement the market shock buffering capacity of female-headed households.

Finally, to safeguard the overall well-being of female-headed households in developing countries against economic shocks, this study specifically calls for enhancing their human and physical capital. The provision of such targeted human and physical capital can mitigate the fallout of economic shocks, such as commodity price hikes and enhance the resilience of female-headed households in developing countries. Coordinated efforts between international donor agencies and national governments can better target such provisions and ensure inclusiveness of female-headed households, who are generally more vulnerable to market shocks.

## Footnotes

1 From June 2006 to June 2008, the wholesale and retail prices of coarse-grain rice, the major food staple of Bangladesh increased by 78% and 82% (FAO and WFP, 2008).

2 In 2005 and 2010 the exchange rate between USD and BDT was USD1 = BDT53.96, USD1 = BDT61.34 USD1 = BDT69.18 (Government of Bangladesh, 2016).

3 2.47105 acres = 1 ha.
